# Incidence of Bacterial Chondronecrosis with Osteomyelitis (Femoral Head Necrosis) Induced by a Model of Skeletal Stress and its Correlation with Subclinical Necrotic Enteritis

**DOI:** 10.3390/microorganisms8020205

**Published:** 2020-02-01

**Authors:** Irene Rojas-Núñez, Ashli F. Moore, A. Gino Lorenzoni

**Affiliations:** Animal Science Department, The Pennsylvania State University, State College, PA 16802, USA; izr66@psu.edu (I.R.-N.); afm4z@virginia.edu (A.F.M.)

**Keywords:** Bacterial chondronecrosis with osteomyelitis (BCO), femoral head necrosis, necrotic enteritis, skeletal stress model, enteritis model

## Abstract

Bacterial chondronecrosis with osteomyelitis (BCO) is a septic necrosis of the skeletal system of unknown origin and an important cause of lameness in broiler chickens. Epithelial inflammation has been proposed as an avenue for bacterial translocation leading to BCO. We evaluated the effect of subclinical necrotic enteritis (SNE), an intestinal inflammatory event, with the development of BCO. In each of two experiments, chickens were divided into three treatments: (1) SNE challenge, including both dietary (wheat- and fish-based diet) and intestinal pathogenic challenges (*Eimeria maxima* and *Clostridium perfringens*), (2) dietary challenge only, and (3) control diet (corn-and soy-based diet). Floor ramps were introduced as part of an established method for increasing the frequency of BCO. The efficacy of the SNE challenge was corroborated by necropsy evaluation of a representative sample of the population. At the end of each experiment, all birds were evaluated for BCO. A high incidence of BCO was found, even in birds with no external signs of lameness. However, the incidence of BCO was not correlated with the intestinal challenge. Conclusions: under the conditions used in these studies, a treatment that is associated with severe damage to the intestinal mucosa does not change the incidence of BCO in broiler chickens.

## 1. Introduction

Lameness is a common condition that affects broiler flocks worldwide. Factors contributing to lameness include infectious agents, genetic selection, growth rate, body conformation, exercise, and nutrition [1,2,3]. Lameness in chickens has a direct impact on their welfare and performance [1,4]. In commercial flocks, 0.5% to 4.0% of birds are culled due to lameness, which has been estimated to account for losses of ~$100 million per year in the United States alone [5]. 

Bacterial chondronecrosis with osteomyelitis (BCO), a bacterial infection with subsequent necrosis of the skeletal tissue, is considered one of the most common skeletal disorders responsible for lameness in meat-type chickens [5,6], affecting ~1% of rapid-growing broilers [6]. The bones most frequently affected by BCO are the femora, tibiae, and fourth thoracic vertebrae [5,6]. The rapid growth of immature bones and excessive torque due to rapid increases in body weight predispose these bones to microfractures and clefts, which can interrupt blood circulation, causing focal ischemia as well as providing an ideal niche for bacterial colonization [6,7]. In the laboratory, mechanical models that create gait instability, which increases torque on the joints of the lower limbs and spine, have been used successfully to increase the frequency of BCO in broilers [6,8,9], making it more tractable for scientific study. 

Previous experiments using live pathogen exposure models have shown that bacteremia is essential for the pathogenesis of BCO; nonetheless, the natural port of entry for the responsible bacteria has not been identified [5]. Since the organisms that have been recovered from the affected bones (*Escherichia coli*, *Staphylococcus aureus*, *S. agnetis*, *Enterococcus cecorum*, *Salmonella enteritidis*) can be routinely isolated from the intestine, the intestinal epithelium is currently the main candidate for the source of bacteria leading to BCO [6,10,11]. Indeed, intestinal exposure and treatment approaches support this idea. Oral inoculation with *E. cecorum* leads to a greater incidence of vertebral BCO in broilers, as compared to air sac or intravenous exposure routes [12], although this effect may be dose-dependent. Administration of *S. agnetis* in the drinking water increases the incidence of BCO in birds subjected to a “wire floor” gait instability model [13]. Moreover, administration of oral probiotics reduces the incidence of BCO [10]. In addition to live pathogen exposure models, BCO can be recreated in the laboratory without the purposeful administration of bacteria [6]. Such models may be a more suitable strategy for mimicking the conditions that generate the disease in production settings. 

Necrotic enteritis (NE), and its milder form, subclinical necrotic enteritis (SNE), are severe multifactorial diseases of chickens, strongly associated with NetB-positive strains of *Clostridium perfringens* [14,15,16], leading to severe macro- and microscopic alterations of the intestinal mucosa. *C. perfringens* releases toxins capable of destroying enterocytes by forming pores on their surface. In addition, the extracellular matrix and tight junctions between enterocytes are disrupted, causing detachment of the epithelium from the basal membrane [15]. NE is associated with depression, inappetence, wet litter, and high flock mortality, often sudden and without preceding clinical signs [14,15]. The reduction in the use of antibiotics has led to a reemergence in this disease, which according to recent estimates, leads to losses of more than $5 billion worldwide [17]. In SNE, the term *subclinical* refers to the fact that the birds usually do not show overt signs of illness and, unlike acute clinical NE, there is no mortality [15]. Despite the lack of external clinical signs, birds with SNE have, by definition [18,19], necrotic patches on the small intestine indicating severe intestinal damage. This form of the disease leads to reduced growth and poor feed conversion [15,17,18], and because SNE often remains undetected, it is thought to be even more costly to the industry than acute NE [17]. 

Several models have been developed to generate SNE in the laboratory. Most utilize a coinfection with coccidia (intestinal parasites) as well as *C. perfringens* [19,20,21,22,23]. Many incorporate a proinflammatory diet comprised primarily of wheat [19,20,21,22,23] and/or fish meal [21,22]. A wheat-based diet has been linked to reduced digestibility and increased mucus production, which provides increased substrate for the growth of *C. perfringens* within the intestinal tract [16,24,25]. In addition, diets high in animal protein, especially fish meal, are believed to support *C. perfringens* with key amino acids needed for its growth [16,25,26,27]. Such models have been able to induce SNE in up to ~60% of animals in controlled laboratory studies [19,20,23,28,29]. 

The magnitude of the damage caused by SNE makes it a good candidate for exploring the associations between intestinal damage, bacterial translocation, and BCO. Despite the economic importance of both SNE and BCO, the potential relationship between these diseases has not been explored. The objective of the current study was to determine the effect of severe intestinal damage wrought by SNE on the frequency of BCO in broiler chickens. To this end, we combined established experimental models for inducing SNE and BCO.

## 2. Materials and Methods

All the animal work described in this study was approved by the Institutional Animal Care and Use Committee (IACUC) at The Pennsylvania State University (protocol number PROTO201899112), and all experiments were performed according to the approved guidelines and regulations.

### 2.1. Animal Experiment Design

#### 2.1.1. Experiment 1

Day-old, Hubbard–Ross 708 male broiler chicks, vaccinated only against Marek’s disease, were purchased from a local hatchery and placed in floor pens on fresh wood shavings at the Poultry Education and Research Center (PERC) at The Pennsylvania State University. On day five, stunted birds were euthanized by cervical dislocation. On day 13, twenty-five percent of the birds were randomly chosen and weighed to estimate the mean body weight. Birds weighing ± 10% of the mean body weight were allocated into three groups (*n* = 65 birds/group); birds outside this range were excluded from the study. The groups were: Challenge Diet + Infected, Challenge Diet, and Control Diet. Each group was placed into a 12 m^2^ floor pen equipped with 2 manual feeders and nipple drinkers. The uninfected animals (Challenge Diet and Control Diet) were housed in a separate but identical room to avoid cross-contamination with coccidia and/or *Clostridium*. To induce BCO without the experimental administration of bacteria, we employed wire ramps [6] as a modification of wire flooring models [8,9]. Briefly, ramps were constructed from wire cloth on a wooden frame; the tallest point of the ramp (apex) had a height of 30 cm and formed a 90-degree angle in relation to a 46-cm base. The long side of the formed triangle (hypotenuse) had a length of 55 cm, which yielded a 30-degree angle in relation to the floor, or a 65% slope calculated as the apex height/base. On day 22, ramps were placed in every pen under the drinker lines such that birds had to climb the ramp to access the drinkers. The experiment was terminated on day 50.

#### 2.1.2. Experiment 2

Day-old, Ross 708 straight-run broiler chicks, vaccinated only against Marek’s disease, were purchased from a local hatchery and placed in floor pens as described in Experiment 1. On day five, stunted birds were euthanized by cervical dislocation. On day 16, chickens were weighed and allocated into three groups (*n* = 55, 55, and 54 animals per group) as described in Experiment 1. On day 17 the wire ramps were placed in every pen under the drinker lines. The experiment was terminated on day 59.

### 2.2. Diets

From days 1 through 26, all treatment groups in both experiments received a commercial corn- and soy-based grower diet formulated to meet or exceed NRC requirements. The Control Diet group was kept on this diet until the end of the experiment. On day 27, the Challenge Diet and Challenge Diet + Infected groups received a wheat- and fish meal-based diet until the end of the experiment. Neither diet was supplemented with antimicrobial growth promotors or anticoccidial products. Both diets were supplemented with sodium bentonite (6 pound/ton) during Experiment 2 to minimize potential effects of mycotoxin contamination. Diet formulations are given in Table 1.

### 2.3. Subclinical Necrotic Enteritis Model

For the Challenge Diet + Infected group, we used dietary and pathogenic treatments to induce subclinical necrotic enteritis (SNE) using a modification of the procedures described in Shojadoost et al. [16] and previously used successfully in our laboratory [29]. Briefly, on day 23, birds from the Challenge Diet + Infected group (Experiment 1, *n* = 65, Experiment 2, *n* = 55) were gavaged with 3000 and 5000 oocysts of *Eimeria maxima*, respectively, diluted in 1 mL of saline solution. On days 27, 28, and 29, three NetB-positive *C. perfringens* strains, collected from field cases, were cultured in thioglycolate liquid media (fluid thioglycolate medium, Dot Scientific Inc., Burton MI, USA), and incubated at 37 °C for 16 h inside an anaerobic chamber. Anaerobiosis was achieved by adding a pouch of an anaerobic gas generating system (AnaeroPack System, Mitsubishi Gas Chemical America, INC., New York, NY, USA) inside the chamber immediately before closing it. After incubation, ten-fold serial dilutions of the broth were plated on brain heart infusion agar (Becton, Dickinson and Company, Franklin Lakes, NJ, USA) and incubated at 37 °C for 24 h under anaerobic conditions. Colony-forming units (CFUs) were counted to determine the experimental dose. 

On days 28, 29, and 30, the feed in the Challenge Diet + Infected group (Experiment 1, *n* = 65, Experiment 2, *n* = 55) was inoculated with *C. perfringens* broth (~ 1 × 10^9^ CFU/bird). Birds were fasted for 8 hours before the first inoculation on day 28. 

### 2.4. Evaluation of Subclinical Necrotic Enteritis

On day 31, nine birds per treatment in Experiment 1 (*n* = 27) and five birds per treatment in Experiment 2 (*n* = 15) were randomly chosen to conduct a direct evaluation of their intestinal tracts. The purpose of this evaluation was to confirm the efficacy of the SNE challenge, which has previously been used in our laboratory and reliably produces ~30–50% of animals with positive SNE scores [29]. The birds were euthanized by cervical dislocation and their intestinal tracts were immediately inspected by a single trained individual. The SNE lesions were scored as follows: intestines with no visible necrosis (score 0); presence of one or two isolated areas of necrosis no larger than 3 mm in diameter (score 1); presence of one or two larger isolated necrotic patches (3–10 mm in diameter, score 2); presence of 3 or more necrotic patches along the length of the small intestine (score 3); presence of multiple (>3) necrotic patches in close succession covering at least 10 cm (lengthwise) of intestine (score 4); coalescent necrotic patches covering at least 10 cm (lengthwise) of intestine (score 5). This scoring system, which has been previously described [29], eliminates the assignment of a positive score for “thin, friable, intestinal walls,” as used in some scoring systems, due to lack of clear association between this lesion and necrotic enteritis [19,29]. For analysis, a bird with an intestinal score of 1 or more was considered positive for SNE, whereas a score of zero indicated that the bird did not have SNE.

### 2.5. Evaluation of Lameness and Changes in Femora and Tibiae

**Figure 1:**
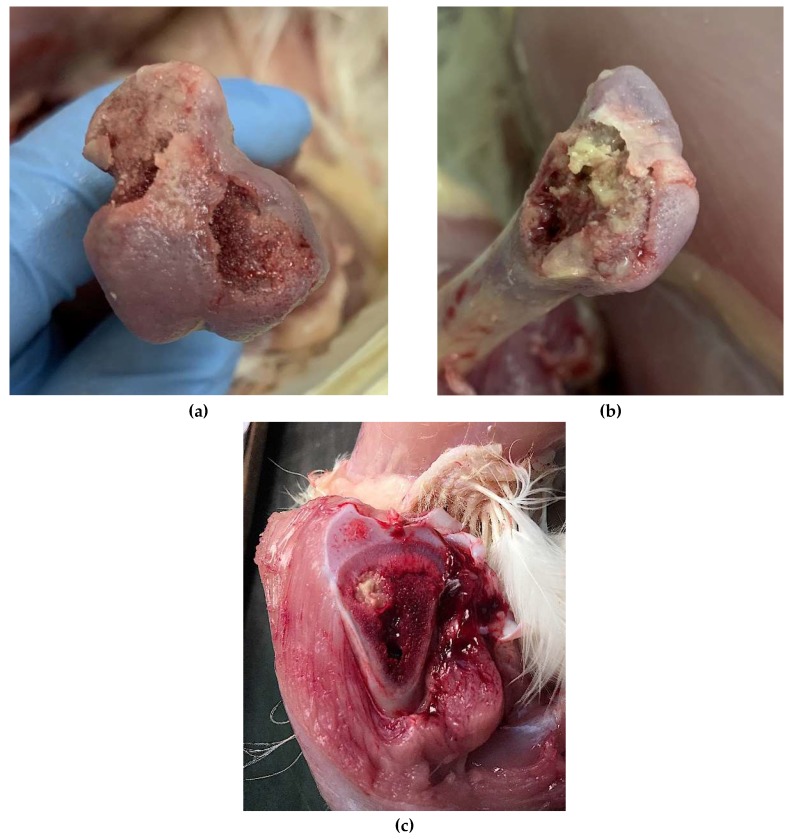
Bacterial chondronecrosis with osteomyelitis classification: (**a**) transitional femoral head chondronecrosis; (**b**) complete femoral head chondronecrosis; (**c**) proximal tibial head chondronecrosis.

From day 31 to the end of the experiments (Days 50 and 59 for Experiments 1 and 2, respectively) all birds were monitored for lameness twice a day by a single trained individual. Birds were considered lame when they either (1) refused to walk, (2) used the tip of one of their wings to stand, or (3) dropped a wing to regain balance while walking. Lame birds were euthanized, and their femora were examined for femoral head chondronecrosis, classified as either complete (upon disarticulation, the femoral epiphysis, physis, and part of the metaphysis remains attached to the acetabulum due to fracturing of the femur; Figure 1 panel b) or transitional (upon disarticulation, some of the femoral epiphysis remains attached to the acetabulum, revealing the underlying surface of the growth plate or physis; Figure 1 panel a). Femora were classified as having cartilage detachment if, upon disarticulation, the layer of hyaline cartilage that covers the growth plate of the femoral head detached from the femoral epiphysis [7]. The tibiae of the euthanized animals were examined for proximal tibial chondronecrosis, defined as large necrotic voids in the metaphysis; Figure 1, panel c [1,6,30]. On days 50 (Experiment 1) and 59 (Experiment 2), all remaining birds were weighed, euthanized, and necropsied, and their femora and tibiae were evaluated as described above. We defined a positive outcome of BCO as a bird that showed complete femoral head necrosis, transitional femoral head necrosis, and/or tibial proximal necrosis. 

### 2.6. Splenic Bacterial Cultures

Spleens were aseptically removed and cultured for the presence of bacteria at the time of intestinal necropsies (day 31) and final necropsies (days 50 and 59 for Experiments 1 and 2, respectively). For the intestinal necropsies, we collected spleens from all birds necropsied. For the final necropsies, we collected spleens from a sample of birds necropsied (*n* = 33, 35, and 32 for Challenge Diet + Infected, Challenge Diet, and Control Diet groups, respectively). Briefly, spleens were homogenized in yeast extract media, plated on Brucella agar with 5% sheep blood (Remel, Lenexa, KS, USA) and MacConkey agar (Difco^TM^, Becton-Dickinson, Franklin Lakes, NJ, USA), and incubated aerobically and anaerobically for 24 h at 37 °C. The presence of bacterial colonies was recorded qualitatively (positive/negative). 

### 2.7. Statistical Analysis

Incidences of SNE and BCO, as well as spleen culture results, were analyzed with nominal logistic regression, with treatment group as the predictor variable and the frequency of binary outcome (yes/no) as the response variable. The Challenge Diet group was used as the reference level for the predictors. For SNE, we employed penalized maximum likelihood (Firth’s bias adjustment) for parameter estimation to correct for rare-event bias. Spleen culture results were additionally analyzed with leg status (BCO or non-BCO) as the predictor variable. Body weights were compared between birds with BCO and birds without BCO using Student’s t-test. All analyses were carried out using the software JMP® (Version Pro 12, SAS Institute Inc., Cary, NC, USA).

## 3. Results

### 3.1. Subclinical Necrotic Enteritis Challenge

In Experiment 1, the feed of the Challenge Diet + Infected group was inoculated with broth containing 1.3 × 10^10^, 4.75 × 10^9^, and 4.5 × 10^7^ CFU/mL of *C. perfringens*, for the first, second, and third inoculations, respectively. In Experiment, 2 the bacterial counts for the first, second, and third clostridial broths used to inoculate the feed of the Challenge Diet + Infected group were 8.5 x 10^8^, 2.07 × 10^7^, and 1.63 × 10^7^ CFU/mL, respectively. 

**Figure 2:**
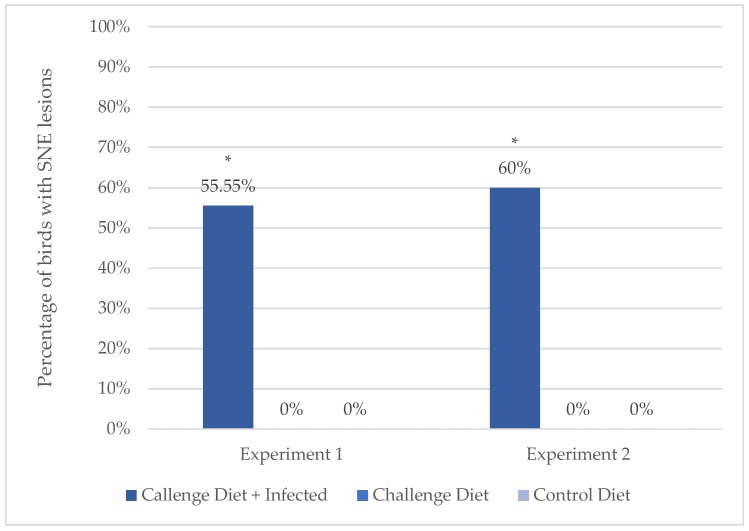
Incidence of subclinical necrotic enteritis (SNE) during Experiments 1 and 2. Randomly selected birds from each treatment group were necropsied at 31 days of age (Experiment 1, 9 birds/group; Experiment 2, 5 birds/group). Asterisks (*) denote statistically significant differences (*p* < 0.05) in the incidence of SNE between the Challenge Diet + Infected and Challenge Diet groups.

In both experiments, the incidence of SNE on day 31 was significantly affected by the treatments (*p* = 0.0029 and *p* = 0.0375, Experiments 1 and 2, respectively; Figure 2). 

#### 3.1.1. Experiment 1

None of the birds showed clinical signs of intestinal disease (e.g., depression, diarrhea, mortality). Five out of the nine birds necropsied on day 31 from the Challenge Diet + Infected group were found positive for SNE, with scores between 1 and 4 (one bird with score 1, two birds with score 2, and two birds with score 4). Birds from the Challenge Diet and Control Diet groups (n = 9 birds/treatment) did not present lesions indicative of SNE. The incidence of SNE significantly differed between Challenge Diet + Infected and Challenge Diet groups (*p* = 0.0006). As expected, no significant difference in the incidence of SNE was found between the Challenge Diet and Control Diet groups (Figure 2).

#### 3.1.2. Experiment 2

None of the birds showed clinical signs of intestinal disease. Three out of the five birds necropsied on day 31 from the Challenge Diet + Infected group were found positive for SNE, with scores between 2 and 3 (two birds with score 2, and one bird with score 3). Birds from the Challenge Diet and Control Diet groups (*n* = 5 birds/group) did not present lesions indicative of SNE. The incidence of SNE significantly differed between the Challenge Diet + Infected and Challenge Diet groups (*p* = 0.01). As expected, no significant difference in the incidence of SNE was found between the Challenge Diet and Control Diet groups (Figure 2).

### 3.2. Evaluation of Femora and Tibiae

#### 3.2.1. Experiment 1

**Figure 3:**
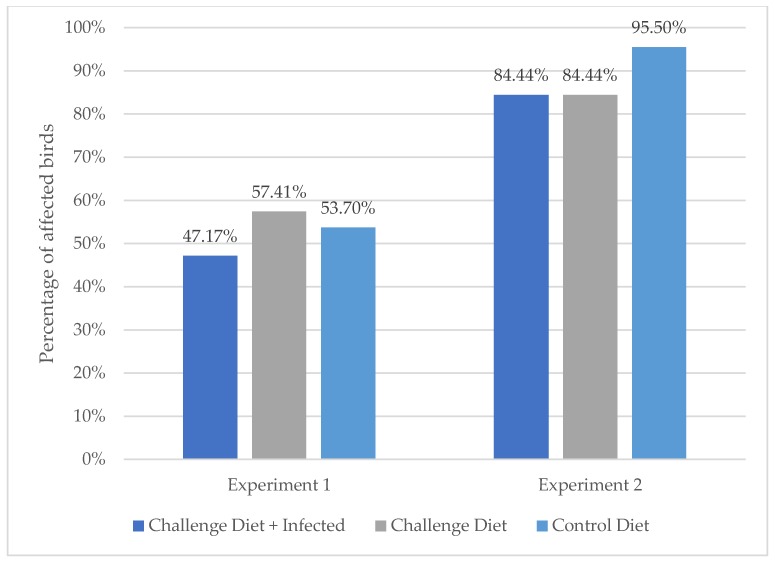
Incidence of bacterial chondronecrosis with osteomyelitis (BCO) for Experiments 1 (day 50) and 2 (day 59). For both experiments, there was no significant effect of treatment on the incidence of BCO.

The results are summarized in Table 2; statistical comparisons are depicted in Figure 3. Complete femoral head BCO was not observed in this experiment. BCO lesions (transitional femoral head BCO and/or tibial BCO) were observed in 25 birds from the Challenge Diet + Infected group (*n* = 53), 31 birds from the Challenge Diet group (*n* = 54), and 29 birds from the Control Diet group (*n* = 54). Transitional femoral head BCO was observed in 25 birds from the Challenge Diet + Infected group (*n* = 53), 31 birds from the Challenge Diet group (*n* = 54), and 29 birds from the Control Diet group (*n* = 54). Tibial BCO was found in one bird from the Challenge Diet group and in one bird from the Control Diet group (these two birds also had transitional femoral head BCO). Out of the 85 birds with BCO, 51 birds presented lesions in only one anatomical site (left femur, right femur, left tibia, or right tibia), and 34 birds presented lesions in two anatomical sites. We found no significant effect of treatment on the incidence of BCO on day 50. Femoral cartilage detachment was the only degenerative change observed in 15 birds from the Challenge Diet + Infected group (*n* = 53), 14 birds from the Challenge Diet group (*n* = 54), and 15 birds from the Control Diet group (*n* = 54). No bodyweight differences were found between birds with BCO and birds without BCO (*p* = 0.267).

#### 3.2.2. Experiment 2

The results are summarized in Table 2 and depicted in Figure 3. BCO lesions (complete and/or transitional femoral head BCO and/or tibial BCO) were observed in 38 birds from the Challenge Diet + Infected group (*n* = 45), 38 birds from the Challenge Diet group (*n* = 45), and 42 birds from the Control Diet group (*n* = 44). Femoral head BCO (complete and/or transitional) was observed in 37 birds from the Challenge Diet + Infected group, 37 birds from the Challenge Diet group, and 40 birds from the Control Diet group. Complete femoral head BCO was observed in one, three, and one birds from the Challenge Diet + Infected, Challenge Diet, and Control Diet groups, respectively. Transitional femoral head BCO was observed in 36 birds from the Challenge Diet + Infected group (*n* = 45), 34 birds from the Challenge Diet group (*n* = 45), and 39 birds from the Control Diet group (*n* = 44). Tibial BCO was observed in nine birds from the Challenge Diet + Infected group (*n* = 45), with eight of them also presenting femoral head BCO; in ten birds from the Challenge Diet group (*n* = 45), with nine of them also presenting femoral head BCO; and in 29 birds from the Control Diet group (n = 44), with 27 of them also presenting femoral head BCO. Femoral cartilage detachment was the only degenerative change observed in one bird from the Challenge Diet + Infected group (*n* = 45) and in one bird from the Challenge Diet group (*n* = 45). Out of 118 birds with BCO 32, 54, 19, and 13 had lesions in one, two, three, and four anatomical sites, respectively. We found no significant effect of treatment on the incidence of BCO on day 59. No bodyweight differences were found between birds with BCO and birds without BCO (*p* = 0.799).

### 3.3. Lameness Incidence 

#### 3.3.1. Experiment 1

No lame birds were observed in this experiment.

#### 3.3.2. Experiment 2

From days 1 to 31, no lame birds were observed. From days 32 to 59, three birds from the Challenge Diet + Infected group were diagnosed with lameness. One of these presented lameness on day 50 and was diagnosed with transitional femoral head BCO on the right leg; the second presented lameness on day 56 and was diagnosed with bilateral transitional femoral head BCO and tibial BCO in the left leg; the third presented lameness on day 59 and was diagnosed with complete femoral head BCO in the right leg and transitional femoral head BCO in the left leg. From days 32 to 59, three birds from the Challenge Diet group were diagnosed with lameness. One of these presented lameness at day 53 and was diagnosed with bilateral complete femoral head and tibial BCO; the second presented lameness on day 55 and was diagnosed with transitional femoral head BCO in the left leg and femoral cartilage detachment in the right leg; the third presented lameness on day 56 and was diagnosed with bilateral transitional femoral head BCO and tibial BCO in the left leg. One bird from the Control Diet group presented lameness on day 53 and was diagnosed with bilateral femoral head and tibial BCO.

In summary, the overall incidence of lameness was 0% (*n* = 195) during Experiment 1 (from days 0 to 50) and 4.37% (*n* = 160) during Experiment 2 (from day 0 to 59). 

### 3.4. Spleen Cultures

Samples from Experiments 1 and 2 were combined for analysis. For the samples taken on day 31, positive spleen cultures were found in 2 out of 14 birds (14.3%) in the Challenge Diet + Infected group and 7 out of 14 (50%) in both the Challenge Diet and Control Diet groups. The effect of treatment group was not significant (*p* = 0.064). For the final necropsies, positive spleen cultures were found in 11 out of 33 birds (33.3%) in the Challenge Diet + Infected group, 22 out of 35 birds (62.9%) in the Challenge Diet group, and 18 out of 32 bird (56.3%) in the Control Diet group. Treatment group had a significant effect on spleen culture outcome (*p* = 0.038), with the Challenge Diet + Infected group having significantly fewer positive spleen cultures than the Challenge Diet group (*p* = 0.015). The Challenge Diet group was not different from the Control Diet group (*p* = 0.44). We did not find a difference in the number of positive spleens when comparing birds with BCO and birds without BCO (28 out of 72 and 12 out of 28, respectively; *p* = 0.72). 

## 4. Discussion

The intestine has been proposed as a source of the bacteremia that is essential for the pathogenesis of BCO [6]. We reasoned that damaging the intestinal epithelial barrier would lead to increased bacterial translocation and thus, increased incidence of BCO. However, we found no correlation between the SNE challenge and incidence of BCO, despite the fact the experimental treatments used for inducing SNE and BCO were both successful. Indeed, the incidence of BCO was high for all treatments in both experiments, indicating that the mechanical stress exerted by the ramps was sufficient to initiate the pathology. Moreover, the intestinal challenge (inflammatory diet, inoculation with *E. maxima* and *C. perfringens*) successfully induced SNE on day 31, with over half of the birds sampled scoring positive for SNE. This is well within the range of the most successful SNE models [19,20,23,28,29]. 

Although there are simpler methods for generating intestinal damage (e.g., chemical challenges), we sought to explore the effects of SNE on BCO due to the relevance of SNE in the poultry industry. SNE is one of the most powerful offenses to the intestinal epithelium that does not cause mortality, and thus allows an examination of BCO lesions weeks after the intestinal insult. Most of the published models for generating NE use coccidia challenges plus dietary interventions or immune suppression to generate the disease [16]. We chose not to use immunosuppressive agents in order to examine the effect of a damaged, but immune-competent, epithelial barrier on the incidence of BCO. Immunosuppression likely alters the process of bacterial translocation independent of direct epithelial damage. Generating SNE in the laboratory is not a straightforward process. Models that utilize *E. maxima* require titration of the parasite dose to produce appropriate intestinal damage, i.e., damage that does not overwhelm the animal but that generates enough inflammation (mucus and plasma protein secretion) to support the growth of *C. perfringens*. In the current study, we used two different parasite doses for Experiments 1 and 2, respectively. The *E. maxima* used in these experiments was kindly donated by Dr. Fitz-Coy (Merck), who suggested the doses based on the pathogenicity exhibited by the strain. 

To increase the susceptibility of birds to develop BCO, we used pens equipped with steep ramps that birds had to climb on a regular basis to access the drinkers [6]. We observed a greater incidence of BCO during Experiment 2 (birds evaluated at 59 days of age) compared to Experiment 1 (birds evaluated at 50 days of age). This is consistent with previous studies [9,10,31,32,33] reporting that, after 30 days of age, the incidence of BCO progressively increases with age up to 62 days. In an attempt to synchronize the timing of possible bacterial translocation with increased susceptibility to skeletal microfractures, we timed the intestinal challenge such that the intestinal lesions typical of SNE would develop after 30 days of age. 

A previous study investigating the effects of intestinal inflammation on bacteremia and BCO reported a reduced incidence of *E. cecorum*-derived spinal BCO when birds were co-infected with *E. cecorum* and coccidia, compared to birds infected only with *E. cecorum* [34]. This study also reported a decrease in *E. cecorum*-positive spleen cultures in the co-infected group. The authors postulated that these results could be explained by “increased non-specific immune surveillance of the intestine”, triggered by coccidia, which could decrease the rate of bacterial translocation from the intestine to the bloodstream and bones [34]. The results of our study are consistent with this hypothesis. We found significantly fewer positive spleen cultures in the Challenge Diet + Infected group than in the Challenge Diet group at the time of final necropsy. A similar trend was seen when the spleens were sampled shortly after the intestinal challenge, although this did not reach statistical significance. It is possible that inoculating coccidia several days before the clostridial challenge (to allow for parasite cycling) allowed enough time for the animals to mount a protective innate immune response that served as an additional barrier to the development of BCO, even in the presence of intestinal necrosis. Indeed, components of the innate immune system, including macrophages and granulocytes, play a role in the early stages of coccidial infection and show a massive infiltration into the lamina propria [35,36]. 

Interestingly, we found no difference in the number of positive spleen cultures when comparing birds with BCO versus birds without BCO. This finding appears to be inconsistent with the prevailing hypothesis that bacteremia precedes BCO [5,6]. However, in order to determine the potential association between BCO and bacterial translocation, we sampled spleens at the final necropsy, weeks after the expected peak of intestinal damage. Bacteria that translocated when the intestines were compromised could have already been eliminated from the spleen. On the other hand, a field study of *E. cecorum*-derived spinal BCO found that bacterial presence in the spleen persisted, with increasing incidence, for several weeks [37]. In general, however, the timing of pathogenic events preceding BCO (translocation, bacteremia, colonization of bone tissue, etc.) remain largely undefined, and it remains to be determined whether or not spleen culture is a suitable measure of bacterial translocation related to BCO in chickens, and if so, the ideal timing of spleen sampling.

In the spleens sampled shortly after the intestinal challenge, there was no relationship between intestinal necrosis and positive spleen culture. This finding is surprising, given previous evidence that NE leads to increased intestinal permeability and bacterial translocation from the intestine to the liver [38]. Clearly, a more extensive evaluation of bacterial translocation and/or bacteremia is necessary to clarify the effect of *C. perfriengens*-mediated intestinal necrosis on the translocation of naturally occurring bacteria (as opposed to experimentally inoculated bacteria). The bacterial species, route and type of administration, as well as methodology for measuring translocation are all important factors to consider.

Moreover, the timing of the intestinal injury could be a critical factor in susceptibility to bacterial translocation. It is possible that young intestines are more susceptible than mature intestines to bacterial leakage in response to mucosal damage. Furthermore, even though lower body weights do not support the development of skeletal microfractures in the ramp model, circulating bacteria may be able to migrate through the fenestrated capillaries of the skeletal system and remain attached to the cartilage matrix of young birds until the microfractures develop, which then provide a perfect niche for bacterial growth at an older age. In fact, using molecular techniques, bacteria can be found in the bones of healthy birds on a regular basis [39]. In our studies, the high incidence of BCO suggests that bacterial translocation occurred at some point in the life of the animals, regardless of the lesions generated by the SNE challenge. It is possible that bacteria translocated prior to the SNE challenge from sources other than the intestinal tract (integument, respiratory tract, contaminated navels) or from multiple sources, supporting a multifactorial pathogenesis for BCO [5,6]. Moreover, vertical transmission of bacteria has also been implicated in some cases of BCO [6], and we cannot rule out this possibility in the current set of experiments. 

In our experiments, the overall incidence of lameness was very low compared to that reported by Wideman [6] using a similar mechanical model (27% lameness). Indeed, the low incidence of lameness observed in Experiment 1 prompted us to extend the duration of Experiment 2, in an effort to reveal any latent lameness. Several factors could explain the variation in the incidence of lameness among studies, including unintentional pathogen exposure (from water lines, litter, hatchery, breeders, farm environment), diet composition (final body weights of the animals), diet contaminants (mycotoxins), and genetic lines of the animals [6,32]. Consistent with other studies [8], we found a large proportion of birds that did not show evident signs of lameness even in the presence of advanced necrotic lesions in their bones, highlighting the relevance of the subclinical presentation of BCO [39]. *Staphylococcus*, *Salmonella*, and *Enterococcus* are among the genera of bacteria that are most commonly isolated from BCO lesions. *Staphylococcus* and *Salmonella* have been documented as food poisoning agents for decades, and there is emerging evidence that *Enterococcus* plays a role in foodborne illnesses via the spread of antibiotic resistance genes [40]. Although efforts to detect BCO noninvasively are underway [41], current technology for BCO detection requires slicing the bones of the carcass, which is not a process conducted routinely in poultry slaughterhouses. This poses a risk that birds with infected bones could reach the processing plant. The relevance for food safety becomes clear when considering that longer cooking times are needed to reach critical pathogen-inactivating temperatures in bones than in soft tissues.

## 5. Conclusions

A subclinical necrotic enteritis challenge at 28–30 days of age did not increase the incidence of BCO in broiler chickens at 50–59 days under the conditions used in our experiments, despite the fact that the SNE challenge was successful and the overall incidence of BCO was rather high. The current study is not without limitations; the models for SNE and BCO are both relatively complex and may not reflect conditions in the field. Furthermore, it may be necessary to increase the sample size and provide additional replication to detect any effect of SNE on BCO, if such a relationship exists. 

## Figures and Tables

**Table 1:** Diet composition for the soy- and fish meal-based diets used in experiments 1 and 2.

Ingredient	% Inclusion (Dry Matter Basis)	
	Control Diet	Challenge Diet
Corn	54.80	22.97
Wheat grain	-	35.05
Fish meal	-	10.00
Soybean	28.10	26.90
Distiller grains with solubles	10.00	-
Soybean oil	3.55	-
Fat–lard	-	3.77
Limestone	1.40	-
Monocalcium phosphate 21%	0.52	0.06
CaCO_3_	-	0.52
Sodium bentonite ^1^	0.30	0.30
Sodium sesquicarbonate	0.25	-
DL-Methionine	0.24	0.04
Lysine sulfate (Biolys) 70	0.24	-
Salt	0.22	0.30
Choline 70%	0.10	-
Threonine	0.07	-
Hy-D premix 62.5	0.05	-
Vit broiler ^2^	0.05	-
Vit premix ^3^	-	0.40
Wengers feed mill trace mineral	0.05	-
Tribasic copper chloride	0.03	-
Alphagal 280 P	0.02	-
Quantum blue phytase (poultry)	0.01	-
Calculated Nutrients		
ME (kcal/kg)	3286.90	3108.04
Crude protein (%)	20.97	23.99
Digestible lysine (%)	1.05	1.43
Digestible methionine (%)	0.52	0.48
Digestible methionine + cystine (%)	0.81	0.83
Digestible threonine (%)	0.73	0.92
Digestible arginine (%)	1.16	1.52
Digestible tryptophan (%)	0.20	0.29
Calcium (%)	0.75	0.84
Available phosphorus (%)	0.41	0.42
Sodium (%)	0.19	0.18

^1^ Included in Experiment 2. ^2^ Supplement composition is a proprietary blend that meets or exceeds all NRC requirements for chicken grower diet. ^3^ Supplement contains: vitamin A 1,200,000 IU/lb, vitamin D3 400,000 IU/lb, vitamin E 3000 IU/lb, riboflavin 800 mg/lb, D-pantothenic acid 1400 mg/lb, niacin 6000 mg/lb, choline 60,690 mg/lb, vitamin B-12 1.6 mg/lb, zinc 2.2046%, iron 1.1023%, manganese 2.6455%, copper 1764 ppm, iodine 220 ppm, selenium 60 ppm. .

**Table 2:** Evaluation of femora and tibiae for Experiments 1 (day 50) and 2 (day 59). CFHBCO = complete femoral head BCO, TFHBCO = transitional femoral head BCO, TBCO = tibial BCO, FCD = femoral cartilage detachment. For each condition, the number of birds afflicted is given, followed by the percentage of birds in parentheses.

Experiment 1						
Treatment	n	BCO	CFHBCO	TFHBCO	TBCO	FCD
Challenge Diet + Infected	53	25 (47.2)	0 (0.0)	25 (47.2)	0 (0.0)	15 (28.3)
Challenge Diet	54	31 (57.4)	0 (0.0)	31 (57.4)	1 (1.8)	14 (25.9)
Control Diet	54	29 (53.7)	0 (0.0)	29 (53.7)	1 (1.8)	15 (27.8)
Experiment 2						
Treatment	n	BCO	CFHBCO	TFHBCO	TBCO	FCD
Challenge Diet + Infected	45	38 (84.4)	1 (2.2)	36 (80.0)	9 (2.0)	1 (2.2)
Challenge Diet	45	38 (84.4)	3 (6.7)	34 (75.5)	10 (22.2)	1 (2.2)
Control Diet	44	42 (95.5)	1 (2.3)	39 (88.6)	29 (65.9)	0 (0.0)
